# Safeguarding marine protected areas from cumulative effects: a review of methods, best practices, and applications

**DOI:** 10.1007/s00267-025-02146-w

**Published:** 2025-03-24

**Authors:** Cathryn C. Murray, Anya Dunham, Emily Rubidge, Fiona T. Francis, Karen L. Hunter, Lucie C. Hannah

**Affiliations:** 1 Institute of Ocean Sciences, Fisheries and Oceans Canada, 9860 West Saanich Road, Sidney, BC Canada; 2 Pacific Biological Station, Fisheries and Oceans Canada, 3190 Hammond Bay Road, Nanaimo, BC Canada

**Keywords:** Cumulative Impacts, Cumulative Effects, Marine Protected Area, Management, Cumulative Effects Assessment

## Abstract

Marine Protected Areas (MPAs) are key ocean conservation tools that can safeguard the diversity and function of marine ecosystems in the face of an increasing footprint and intensity of human activities. To be effective, MPA design, implementation, and management must consider not only individual, but also cumulative effects of historical, current and foreseeable future activities both within and outside MPA boundaries. Cumulative effects are seldom incorporated into MPA management as it can be challenging for MPA practitioners to select appropriate methods of assessment and integration. This paper examines two aspects of cumulative effects related to MPAs: a review of how cumulative effects are currently considered in MPA management worldwide, and a review of the primary and grey literature addressing cumulative effects knowledge and application in MPA contexts. The review of 646 global MPA management plans revealed that 36% did not contain any cumulative effects-related search terms and therefore likely lacked any provisions for, or even mentions of, cumulative effects. The review of cumulative effects knowledge found that few projects included all cumulative effects steps: scope and structure, assessment, and decision-making. Although significant advances have occurred in risk-based and spatial cumulative effects assessment methods over time, decision-making is rarely included in any cumulative effects projects. To bridge the gap between theory and practice, we propose a framework that embeds cumulative effects within the MPA designation and adaptive management process which will enable comprehensive scoping, meaningful assessments, and clear and transparent decision-making with respect to cumulative effects.

## Highlights

- Marine Protected Area (MPA) design, implementation, and management must consider not only individual, but also cumulative effects
- A review of 646 global MPA management plans revealed that 36% did not contain any cumulative effects-related search terms
- Few MPA projects included all three steps of the cumulative effects framework: scope and structure, assessment, and decision-making
- Significant advances have occurred in risk-based and spatial cumulative effects assessment methods over time, however results are rarely integrated into the decision-making step
- To bridge the gap between theory and practice, we propose a framework that fully embeds cumulative effects within the MPA designation and adaptive management process

## Introduction

Marine Protected Areas (MPAs) are recognized as a powerful tool for conserving marine resources and maintaining ecosystem resilience (e.g., Ban et al. 2019; Edgar et al. 2014). However, the effectiveness and function of MPAs around the world is threatened by diverse human activities, and managers are faced with the increasingly difficult and complex task of determining how to best protect these areas (Batista et al. 2014; Zentner et al. 2023; Zupan et al. 2018). MPA managers must not only restrict activities occurring within the boundaries, traditionally fishing, but may also need to carefully manage the MPA in the context of exogenous pressures, some of which may be outside their management control. The threats posed by global climate stressors, land-based and coastal activities, as well as persistent pollutants highlight that cumulative effects are a concern for MPAs even under the strictest protection standards. Cumulative effects (CE) are commonly defined as the changes in the environment caused by interactions among human activities and natural processes accumulating over space and time (CCME, 2014). While cumulative effects assessment is firmly embedded within impact assessment (Blakley and Russell, 2022; Jones, 2016; Noble, 2015) it has rarely been practically applied in other marine management realms.

Few jurisdictions around the world include the consideration of cumulative effects as an explicit part of MPA management (Hollarsmith et al. 2022; Kirkfeldt and Andersen, 2021; Willsteed et al. 2023). In the United States, assessment of CE is required in the decision-making process for federal actions under the National Environmental Policy Act (40 CFR 1508.7), yet clear guidance on how to do so is absent. The MPA network established under California state’s Marine Life Protection Act requires managers to take into account stressors that impact these areas and their cumulative effects, although the term is not explicitly stated (Gleason et al. 2013). In the European Union (EU), member states implement the EU Marine Spatial Planning Directive (MSPD) requiring them to assess impacts of pressures and develop measures leading to good environmental status (Korpinen et al. 2013). However, this policy lacks clear guidance on CE so there have been only a few examples of EU member states using CE assessment in the EU MSPD processes (Kirkfeldt and Andersen, 2021). In Canada, regulations developed under the Oceans Act (S.C. 1996, c. 31) for individual MPAs include requirements for Ministers to consider cumulative effects during the approval of activity plans, yet there is currently no standardized process to apply the consideration.

In concert with the escalating management need to consider cumulative effects in decision making, knowledge and scientific research into cumulative effects continue to progress and evolve (Noble, 2015; Curren, 2019; Blakley, 2021). The rapidly developing field of cumulative effects assessment has the potential to support and guide MPA managers in their efforts, using a structured approach to evaluate the collective effects of past, present, and future activities affecting an MPA. Knowledge for incorporating CE in MPA management can be organized around three steps: (i) *scoping and structuring*, (ii) *assessment*, and (iii) *decision-making* (Hollarsmith et al. 2022). Scoping requires gathering and screening information and data for a CE assessment process, including defining the scope, scale, objectives, and parameters. The assessment step characterizes the relationships between components in an evaluation of the individual and/or cumulative effects in the study system. The decision-making step seeks to weigh and choose between management options, balancing environmental, social, and economic priorities. Ideally, the decision-making process is iterative with continued monitoring that can be used to update assessments and revise decisions. The three steps need to be based on defensible evidence, aligned with the overall goal of the process, and yet be straightforward and timely due to the often-constrained timelines of decision-making processes. This is challenging given the lack of clear pathways for incorporating CE science outputs into the management decision-making process (Willsteed et al. 2023). A diversity of CE approaches and methodologies are available, from conceptual models (Knights et al. 2015; Stelzenmüller et al. 2010) to spatial analytical models (Halpern et al. 2008), risk-based approaches (RBA) (Levin et al. 2009; O et al. 2015; Stelzenmüller et al. 2018; Verling et al. 2021), and the application of multiple models in combination (Murray et al. 2020), depending on the objectives and goals of management. However, there is currently no guidance on how to incorporate CE approaches into the regulatory process of MPA establishment and the ongoing management cycle.

The MPA establishment process may vary across legislative tools and jurisdictions, but generally follows a series of phases that start with area identification, the production of an overview report or feasibility study, the development of regulatory intent and stakeholder engagement, and then after MPA establishment, moves into the adaptive MPA management cycle. The challenges and best practices in the field of cumulative effects are well documented, often following lessons learned from cumulative effects assessment associated with environmental impact assessment (Blakley and Russell, 2022; Clarke Murray et al. 2014; Duinker et al. 2013; Foley et al. 2017; Hollarsmith et al. 2022; Noble, 2015; Willsteed et al. 2023). Fundamentally, a shift to a cumulative mindset is required where the practitioner considers the full spectrum of activities, stressors and range of impacts, both in isolation and combined (Judd et al. 2015; Willsteed et al. 2023). Stressors are known to interact, and these interactions are difficult to predict and can complicate management actions so that investigation of interaction types can be informative (Crain et al. 2008; Darling and Côté, 2008). Choosing an appropriate spatial scale and temporal baseline is important to produce meaningful and useful results but often defaults to jurisdictional boundaries and present day (Foley et al. 2017; Hollarsmith et al. 2022; Korpinen and Andersen, 2016). Finally, data availability will be an enduring challenge so that the quantification and presentation of uncertainty is necessary for evidence-based decision-making (Willsteed et al. 2023).

Despite the broad acknowledgement of their importance, the consideration of cumulative effects is seldom implemented in management applications (Hollarsmith et al. 2022; Willsteed et al. 2023). While MPA managers and practitioners can draw upon the growing scientific literature, it remains a challenge to identify, gather and synthesize this information in order to implement appropriate methods in MPA management. Here, we first examine how cumulative effects are currently considered in practice by reviewing a global set of MPA management plans. To help bridge the gap between theory and the practical implementation, we examine the theoretical knowledge base through a review of the primary and grey literature on CE knowledge and theory in MPA contexts. Finally, we apply this knowledge base to advance cumulative effects inclusion in MPA management by proposing a framework to embed CE practices firmly within the MPA management cycle.

## Cumulative Effects Practice in Global MPAs

To explore how cumulative effects are currently considered in MPA management, we utilized a dataset of 646 global MPA management plans (1980–2019) collated by Dunham et al. (2024) and merged into a *text corpus* using a pdftools package for the statistical software R version 4.0.2 (Ooms, 2020; R Core Team, 2020) by O’Regan et al. (2021). All plans included were produced by a legally mandated organization or government authority and pertained to conservation management of MPAs with English as an official language of the parent nation. The plans were automatically searched for terms explicitly related to cumulative effects (“cumulative effects”, “additive effects”, “collective effects”, “combined effects”, “multiple effects”, “cumulative impacts”, “additive impacts”, “collective impacts”, “combined impacts”, “multiple impacts”, “cumulative pressures”, “additive pressures”, “collective pressures”, “combined pressures”, “multiple pressures”) as well as single terms that can be plausibly used to describe CEs (“cumulative”, “additive”, “combined”, “in combination”) using text analysis in R (package “tm”; Feinerer et al. 2008). The search terms were chosen based on the wording encountered in the literature review and a manual review of a subset of randomly chosen management plans from Canada, UK, USA, and Oceania (10 plans from each region) to account for possible differences in terminology between jurisdictions. It is important to note that explicit mention of cumulative effect terms or the number of times a term is mentioned do not necessarily reflect the extent to which CEs are taken into consideration in the management of the respective MPA; in other words, the numbers simply denote term inclusion and not how actionable or operational each management plan or group of plans are when it comes to CEs.

**Fig. 1:**
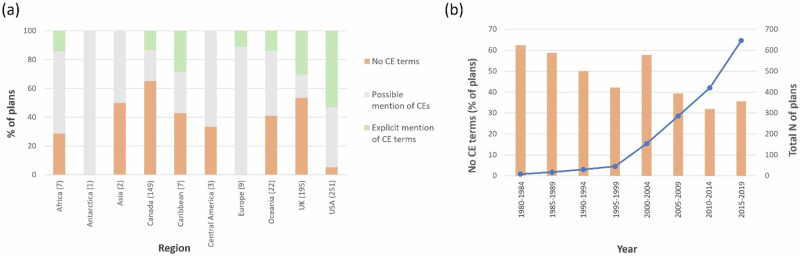
The inclusion of cumulative effects-related terms in MPA management plans by region (**a**) and over time (**b**). “Explicit mention of CE terms” category includes plans that contain terms explicitly related to CEs; “possible mentions of CEs” category includes plans that did not contain explicit CE terms listed above but did contain at least one term that might be related to CEs; “CE terms absent” category refers to plans that did not contain any of our search terms. Blue line represents the cumulative total number of MPA plans over time

Of the 646 global MPA management plans searched, 219 plans (34%) included explicit cumulative effect-related term(s); of those 219 plans, 97 mentioned one term and only once. Of all jurisdictions, the USA had the highest percentage of plans that explicitly included CE terms (53%; Fig. 1a). The highest number of mentions per plan was 379 in the Olympic National Park General Management Plan; with 256 mentions of “cumulative effects”, 122 of “cumulative impacts”, one of “combined effects” (Olympic National Park, 2007), followed by 278 in the Biscayne National Park General Management Plan 2015; 28 mentions of “cumulative effects” and 250 of “cumulative impacts” (Biscayne National Park, 2015); both are detailed plans from the USA.

Two hundred and thirty (36%) of the 646 global management plans did not contain any of our search terms, including single-word ones such as “cumulative”, “combined”, or “multiple”; it is therefore likely that these plans did not include any provisions for, or mentions of, cumulative effects. The highest percentage of plans without a single CE mention was found in Canada (65% of all Canadian plans reviewed), followed by the UK (53%) (Fig. 1a).

Over the study period, the total number of MPA management plans increased from 8 in 1984 to 646 in 2019; the percentage of plans that did not mention any CE-related terms generally decreased over time (Fig. 1b). However, during the two periods of sharper increase in the number of plans developed—1999–2004 and 2014–2019—a concurrent relative increase in plans that did not include CE terms was observed (Fig. 1b): the number of such plans jumped from 42% to 58% and from 32% to 36%, respectively.

As expected, the CE terminology differed between jurisdictions. Of the three regions with the most plans—Canada, UK, and USA—the USA demonstrated the most variety in terminology (Table 1). The terms “cumulative impacts” and “cumulative effects” were, by far, the most frequently used terms in all three countries (Table 1).

**Table 1:** The number of mentions of cumulative effect-related terms in MPA management plans from the three countries with the highest number of plans obtained: Canada (149 plans total, 20 with CE terms), UK (195 plans total, 59 with CE terms), and USA (251 plans total, 133 with CE terms)

		Search term^a^ used									
		cumulative effects	cumulative impacts	additive effects	additive impacts	combined effects	combined impacts	combined pressures	multiple effects	collective impacts	Total number of mentions of cumulative effects terms across plans
**Country**	Canada	38	15	0	0	0	0	0	0	0	53
	UK	59	10	0	39	7	1	0	0	0	116
	USA	1061	2035	7	1	65	5	2	3	1	3180

^a^The terms “cumulative pressures”, “additive pressures”, “multiple pressures”, “multiple impacts”, “collective pressures”, and “collective effects” were not mentioned in any of the plans from these three countries

## Cumulative Effects Knowledge

A structured literature review was conducted to collate projects that consider cumulative effects in MPAs to inform and support the development of a standard framework for integrating cumulative effects assessment into MPA management processes. Web of Science and Google scholar databases were searched using: ALL (fields) = (Marine protected area OR marine protected area network OR marine reserve OR MPA OR MPAn) AND TS = (marine* NEAR management) AND TS = (marine* NEAR design). Searches were carried out using one of the following terms applied separately each time: cumulative effect*, cumulative, multi* stressor*, multi* impact*, multi* human*, human impact*, human activit*. A separate search for ALL(fields) = “cumulative effect*” OR “cumulative impact*” was also done in Web of Science as well as the Federal Science Libraries Network to identify grey literature such as government reports. Titles and abstracts were evaluated to identify relevant articles for inclusion, including articles cited within those identified. The literature search identified 75 relevant articles for further analysis which were grouped into 55 projects as some articles produced by the same research group built on older publications using the same methods (Online Resource 1).

We examined articles on the 55 projects for attributes grouped into the following three categories: 1) Descriptive, 2) CE Framework, and 3) Analytical. Descriptive attributes capture general project information including: (i) Project location; (ii) Authors and publications; (iii) Agency (Government, Academia, Non-Governmental Organization); and (iv) Driver (Marine spatial planning, MPA design and/or management, Impact assessment and/or permitting, and Research). Cumulative Effects Framework attributes indicate which CE framework method each project used (Scope/Structure, Assessment, and Decision-making). Analytical attributes summarized more detailed information on a subset of projects identified in the Framework category as Assessments using the following sections: (i) Assessment type (Risk-based, Spatial, and Spatial & Risk); and (ii) Stressor interaction type (Additive, Antagonistic, Synergistic, Other).

### Descriptive Attributes

**Fig. 2:**
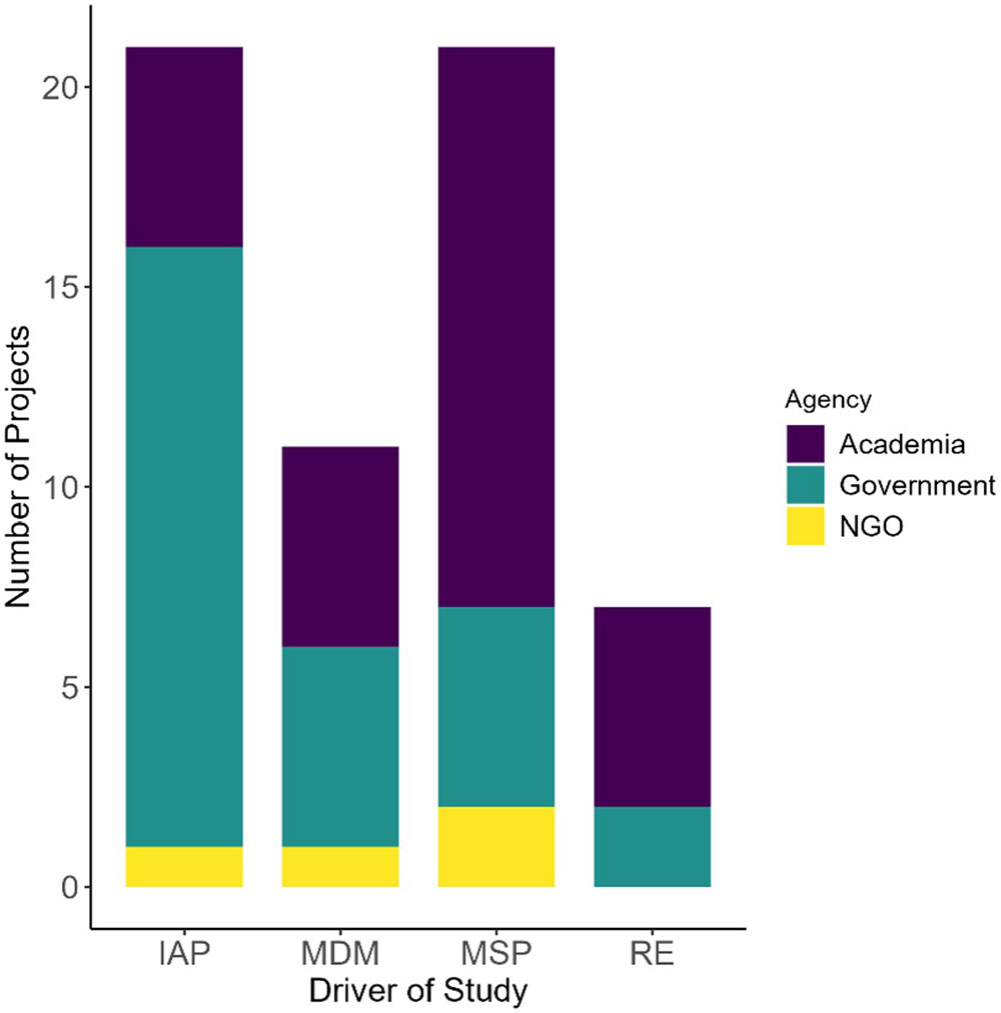
The relative contribution of academia, government and non-governmental organizations (NGO) to the identified projects, grouped by the driver of study (IAP Impact Assessment/Permitting, MDM MPA Design and Management, MSP Marine Spatial Planning, RE Research)

The global distribution of project locations included six regions, with Europe being the most common (49%), followed by North America (34.5%) and Australasia (7%). There were roughly equal contributions of government (49.1%) and academic (52.7%) agencies conducting the work, followed by non-governmental organizations (7.3%) (% add to > 100% because some projects were a collaboration between agencies; for example, 7.1% had contributions from both government and academia) (Fig. 2). The majority of the 55 projects were focused on impact assessment or activity permitting (55%) and marine spatial planning (42%), with the remainder focused on MPA design and management (36%) and on academic research (27%). A small number of projects were focused literature reviews (13%).

### Framework Attributes

**Fig. 3:**
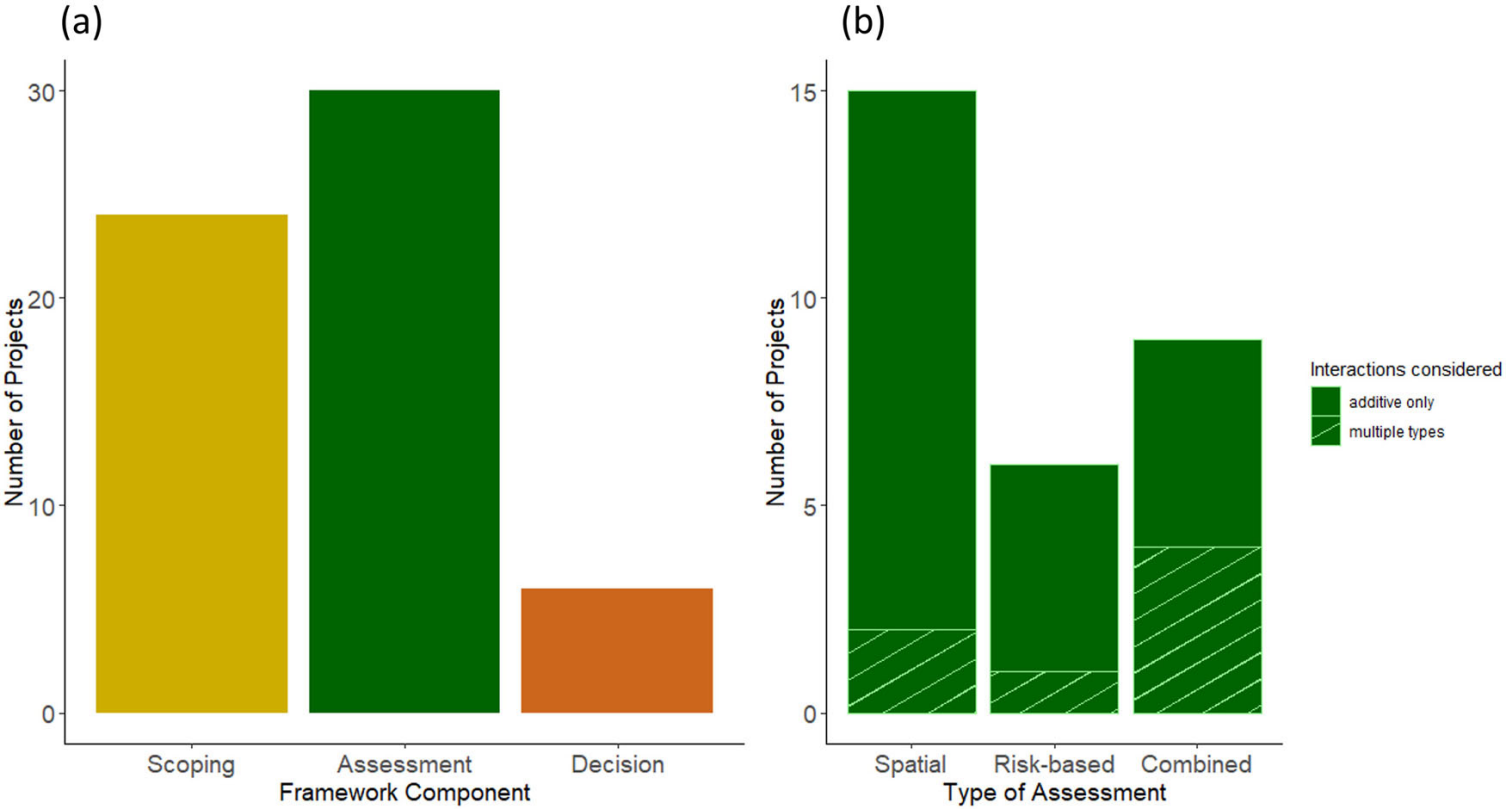
The number of projects that contain (**a**) each of the three cumulative effects steps (Scope and structure, Assessment, Decision-making); some projects contained more than one component. For those projects including an assessment step, (**b**) analytical attributes (spatial, risk-based or a combination of both) and interaction type were considered (additive (unshaded bars) or additional interaction types such as synergistic or antagonistic interactions (hashed bars))

The subset of projects with CE framework attributes (*n* = 35) were evaluated to determine if they contained methods for the three fundamental cumulative effects steps (Scope and structure, Assessment, and Decision-making) (Fig. 3A). Most projects included one or two steps, with assessment being the most commonly included (30 projects, 85.7%), followed by scope and structure (68.6%). Decision-making was the least common step (17.1%). A small number of projects included all three framework components (11.4%), and 13 of the 35 projects (37.1%) included two components (Online Resource 1).

#### Scope and structure

The scoping step of a project is a critical component that involves defining the goals and objectives, the study area, the temporal range, and the endpoints or components of interest. Scoping a cumulative effects study involves gathering and synthesizing complex and wide-ranging information, and this was most commonly done with a standard literature review (Table 2). More recent work has introduced rigorous structured methods for approaching literature reviews, to ensure they are thorough, manageable and comprehensive, for example, literature mapping (Willsteed et al. 2023) or structured literature search (Uthicke et al. 2016). Expert elicitation was used by several projects, with some collecting information on activities and stressors through a combination of literature review and expert feedback gained from workshops (Afflerbach et al. 2017) or conferences (Harris et al. 2015).

**Table 2:** Methods identified in the literature for the scope and structure step of the cumulative effects framework

Method	Description
Review	The purpose, objectives, scale and focus for the assessment can be defined by gathering information through structured *literature review* methods (Uthicke et al. 2016), such as systematic literature mapping (James et al. 2016; Willsteed et al. 2023), or *expert elicitation* methods (Agostini et al. 2015; Álvarez-Berastegui et al. 2014; Anthony et al. 2013).
Linkage networks	The *Driver-Pressure-State-Impact-Response (DPSIR) framework* shows the cause-effect relationships, called impact chains, among the component categories. It was originally developed for aquatic ecosystem management (EEA, 1995; OECD, 1993) and has since been modified (e.g., DAPSIR, DAPSIWRM) and applied in various forms to MPAs (e.g. Menegon et al. 2018; Piet et al. 2021), and by marine management bodies like OSPAR (a cooperative mechanism for 15 governments and the EU to protect the NE Atlantic marine environment) and the EU Marine Strategy Framework Directive (MSFD).
	*Pathways of Effects (PoE)* conceptual models are used to organize knowledge on activities, stressors, and potential effects in a structured, functional, and visual way (Government of Canada, 2012; Hannah et al. 2020). The diagrams visually represent pathway linkages from activities through stressors to effects, and tables of supporting evidence provide background information for those linkages to describe the knowledge available for each linkage from published literature and other sources (DFO, 2014; Gendreau et al. 2018; Giguère et al. 2011; Hannah et al. 2020; Isaacman and Daborn, 2011; Stephenson and Hartwig, 2009).
	*Bow-tie analysis* illustrates pathways between causal factors and the consequences of a central hazard, including the preventative and mitigative management measures, or controls. The intention is to combine bow-tie diagrams for each hazard by identifying linkages between related parameters across all indicator diagrams and visualizing the network of bow-ties. A modified bow-tie analysis (ICES, 2014) is being used in the OSPAR Quality Status Reporting process (OSPAR, 2023).
	*Sankey diagrams and Alluvial plots* are visual tools that illustrate the pathways (links) between connected values (nodes). The height of each node (represented as a vertical line) indicates count/volume, links between nodes have curved lines, the thickness of which links to node height but also indicates count/volume. Sankey diagrams have been used to show relationships among activities and pressures (Fernandes et al. 2020) and Alluvial plots have been used to illustrate the frequencies of the relationships between CEA drivers, case studies, human activities, human pressures, and assessment endpoints (Stelzenmüller et al. 2020), though not for scoping an assessment in this case), and to illustrate magnitude in change in the impact of pressures on ecosystem components under different scenarios in (Hammar et al. 2020).
Screening	The *appropriateness* step in the California Department of Fish and Wildlife framework for decision support on permitting scientific activities in MPAs determines if the proposed scientific activities are appropriate to consider permitting in an MPA (Saarman et al. 2017; Saarman et al. 2018). The UK Government Habitats regulation assessment has a *pre-assessment screening* step that removes the requirement for an assessment if the proposal is determined to be unlikely to have a significant effect on the conservation objectives of the site (either alone or combined) (DEFRA, 2021).

Although information gathered during scoping was often structured into a simple table form (e.g., Ban et al. 2010; Hammar et al. 2020; Mach et al. 2017), in some cases it was also organized by visualizing the connections between a hierarchy of components (Dunstan et al. 2022; Hannah et al. 2020; Hayes et al. 2021; Menegon et al. 2018; Murray et al. 2019; Murray et al. 2021; Piet et al. 2021; Robinson et al. 2014; Uthicke et al. 2016). We will refer to this class of scoping and structuring tools as “linkage networks”, a term comparable to the linkage ‘frameworks’ term used in the EU (Koss et al. 2011; Robinson and Culhane, 2020), that encompasses Driver-Pressure-State-Impact-Response (DPSIR) and Pathways of Effects (PoE) conceptual models, Sankey/Alluvial diagrams, and Bow-tie diagrams (Table 2). Some studies used a screening or pre-assessment step to determine if assessment were required in the MPA (Saarman et al. 2017; Saarman et al. 2018).

#### Assessment

Spatial and risk-based CE assessment methods have continued to be developed, tested and refined since their emergence in the late 2000s, and over time an increasing number of methods that incorporate aspects of both approaches have been published. Our review identified 30 projects with an assessment step; most used spatial mapping (15 projects) or risk-based methods (6 projects), with some using a combination of both types (9 projects) (Table 3; Fig. 5). Spatial mapping assessments have come to be dominated by cumulative impact mapping (Table 3), a method introduced by Halpern et al. (2008). In this stream of research, a map of cumulative impact scores is derived using location and relative intensity of activities, stressors, and their relative impact on components of interest. Risk-based methods evaluate some combination of the dimensions of risk, exposure and consequence, which can be divided further into variables and combined mathematically or statistically (U.S. Environmental Protection Agency 1992). Risk based approaches have been developed to focus project-based assessments on higher risk activities and stressors where resources and/or time are limited (e.g. risk retirement process: Copping et al. 2015; 2020).

**Table 3:** Selected methods identified in the literature for the assessment step of the cumulative effects framework

Method	Description
**Risk-based**	The *Ecological Risk Assessment Framework (ERAF)* is an area-based cumulative effects assessment framework developed specifically for MPAs (O et al. 2015) with three phases: scoping, screening, and risk assessment. The risk assessment includes methods for a qualitative, semi-quantitative, or fully quantitative assessment, and has been applied to three MPAs (Hannah et al. 2019; Rubidge et al. 2018; Thornborough et al. 2018). Fully quantitative risk assessment builds upon Judd et al. (2015), which determines risk by scoring five criteria (Robinson et al. 2013; Piet et al. 2015; Piet et al. 2021; Robinson et al. 2014).
	*Options for Delivering Ecosystem-based Marine Management (ODEMM) Pressure Assessment* method is an ecological risk assessment approach from the European Commission (Pedreschi et al. 2023). It consists of two components: a linkage framework with sector-pressure-ecosystem component linkage chains, followed by a pressure assessment where each individual linkage chain is scored on five attributes; spatial overlap, frequency of occurrence, degree of impact, resilience and persistence. Scoring is consensus-based and completed by a combination of expert opinion and literature review. The results allow a relative ranking of the sectors, pressures, or ecological components in the assessment using summary statistics such as the average or sum of scores (Robinson and Culhane, 2020; Robinson et al. 2014; Robinson et al. 2013).
	The *Comprehensive Assessment of Risk to Ecosystems (CARE) framework* developed in Portugal, is designed to be simple and rapid to use, relying on expert knowledge and minimal background research suitable for data-limited ecosystem assessments (Battista et al. 2017). It differs to most of the other risk-based methods, as it is not hierarchical, and allows for a single analysis of a target to be completed on a single Excel worksheet. This method produces simple threat rankings (low, medium, high) based on a multiplicative approach (similar to the ERAF above).
**Spatial**	*Cumulative Impact Mapping (CIM)* method is the most widely used spatial assessment method; first developed by (Halpern et al. 2009; Halpern et al. 2008) to produce a map of cumulative impact scores. The cumulative impact score is derived using location and relative intensity of activities, stressors resulting from activities, and the relative impact of stressors obtained through expert elicitation or literature review. Though usually applied at large scales, the method has been used or adapted for regional-level applications (Ban et al. 2010) and the MPA network scale (Mach et al. 2017). The method has also been adapted to focus on impacts to single species rather than habitats (Marcotte et al. 2015; Maxwell et al. 2013) and to include temporal (seasonal) changes in stressor impact (Afflerbach et al. 2017).
**Spatial** **& Risk**	The *Cumulative Impacts Supporting Environmental Decisions (CISDM) model* combines qualitative models to identify Driver-Activity-Pressure-Impacts linkages (Anthony et al. 2013). Qualitative models are developed in workshops with scientific experts and then transformed to probabilistic ecosystem models using Bayesian networks. Cumulative effects are assessed by analyzing these models together with spatial zones of influence to calculate estimated risk. Developed to be integrated into the CISDM, the *Spatial Cumulative Impacts Risk Analysis (SCIRA) model* is a combination of risk and spatially-explicit cumulative effects assessments (Uthicke et al. 2016); producing a dynamic spatial predictive model of cumulative impacts (Uthicke et al. 2016). SCIRA uses dynamic environmental layers as input variables, and bathymetry, land, and habitat masks as base layers to produce cumulative-pressure scores as well as ecosystem maps.
	The *Monitoring, Evaluation, Reporting and Improvement (MERI)* framework uses spatial cumulative impact maps to support and visualize results of risk-based assessments and connects to prioritization decisions related to monitoring. Cumulative effects are assessed using a large interaction matrix scored by experts. Vulnerability of ecosystems was determined through expert surveys and was used to weight impact scores (Dunstan et al. 2022; Hayes et al. 2021).

The assessment of cumulative effects can be complicated by the interacting effects of multiple stressors; where additive interactions simply sum the single effects, synergistic interactions magnify the effects, and antagonistic interactions dampen the overall effects (Crain et al. 2008; Darling and Cote, 2008). For most assessments, additive stressor interactions were the only type of interaction considered (76.6%) (Fig. 3B). Antagonistic and synergistic interactions were only used in addition to additive interactions in 23% and 20% of studies, respectively. However, over time, adaptations of the cumulative impact mapping have included non-additive stressor interactions (Furlan et al. 2019). Cumulative impact mapping has further evolved to better suit study requirements (Tulloch et al. 2020), seasonality (Afflerbach et al. 2017), available resources, study area size (Harris et al. 2015), data types (Andersen et al. 2017; Menegon et al. 2018), and data availability (Fernandes et al. 2017; Fernandes et al. 2020).

#### Decision-making

The majority of the projects reviewed did not include clear decision-making methods or explicit links to decision-making processes. There were only six projects out of 35 with decision-making components and they all varied in approach considerably. The pattern of a gradual increase in decision-making methods in assessments over time reflect that this is still an emerging area of research despite being first noted in the literature in 2012, in contrast with the steeper increase seen in assessment methods published over the same period. Decision-making methods need to connect to assessment outputs and link to management objectives in the scoping step to allow for effective testing of management scenarios and thresholds. The methods used can be grouped into structured decision-making, scenario evaluation, probabilistic, and threshold-based methods (Table 4).

**Table 4:** Methods identified in the literature for the decision-making step of the cumulative effects framework

Method	Description
Structured decision making	*Structured Decision Making (SDM)* separates causal and value judgement tasks to minimize common problems with risk-based decision making (Anthony et al. 2013). Embedded in the CISDM, SDM consists of two phases i) identify options and ii) decision analysis (Anthony et al. 2013; Uthicke et al. 2016).
Scenario evaluation	The decision-making *Integrated* *Management Strategy Evaluation (iMSE)* examines how linkage chains (based on Driver-Pressure-State combinations) contribute to the risk of not fulfilling policy objectives, with a reduction in ecological risk associated with a management action reflecting its effectiveness. Effectiveness of a measure reflects the number of impact chains it targets, the weighting of impact chains (based on risk criteria) and the likelihood of the management measure lowering their impact (Piet et al. 2015). This is supported with geospatial visualizations of expected outcomes of management action (Robinson et al. 2014).
Probabilistic	CISDM assessment outputs provides qualitative models and *Bayesian probabilistic models* that represent impact chains. Impact chains form part of the decision-making structure and clear guidance is provided through the use of objectives tabulated against management options and decision making includes examination of management interventions, consequences and tradeoffs (Anthony et al. 2013; Uthicke et al. 2016). Furlan et al. (2020) describe a GIS- based Bayesian Network conceptual model that incorporates probabilities to predict cumulative effects and allows users to alter the probabilities associated with different nodes of the model and examine the changes in cumulative effects with different scenarios.
Threshold-based	*Impact threshold* comparison aims to determine if estimated cumulative impacts of all projects exceed policy-based acceptable impact thresholds (Saarman et al. 2017; Saarman et al. 2018). During threshold setting, managers can consider the proposed activity in context with other activities and influences such as natural climate cycles and extractive activities. The decision component consists of an impact threshold comparison to determine if the estimated cumulative impacts of all projects exceed policy-based acceptable impact thresholds for species, assemblages or habitats.

## Advancing MPA Management

### Cumulative effects are rarely considered in MPA management

Despite the legal requirements to include cumulative effects in MPA management identified by governments around the world, effective practical implementation appears to be lacking across the jurisdictions examined in this study. The majority of MPA management plans examined either do not mention cumulative effects or mention them only once without recommending or outlining the intent for ongoing assessments. Over time (1980–2019), the percentage of plans with no mention of cumulative effects decreased, possibly signifying an improved understanding of its importance. However, during the two periods of sharper increase in the number of MPA plans developed (1999–2004 and 2014–2019), a concurrent relative increase in plans that did not include CE terms was observed; this might reflect limited time and resources hampering careful consideration of CE (and perhaps other aspects) during management plan development.

We must note that the lack of explicit cumulative effects language in the management plans examined does not necessarily signal complete lack of consideration. In some jurisdictions, other mechanisms and processes outside of MPA management plans may be in place to address cumulative effects, such as project-by-project environmental assessment. The MPA management may rely on other management processes, such as Environmental Impact Assessment, where Cumulative Effects Assessment (CEA) is used to determine the potential effects of proposed projects, although the application of CEA in that process has been criticized (Foley et al. 2017; Murray et al. 2018). In addition, some of the management plans our study identified as lacking explicit cumulative effect-related terms may be using terms not picked up by our search method. Finally, our search was limited to plans written in English. Thus, similar to other studies (e.g. Nelson and Shirley, 2023), our search methodology precludes in-depth analysis beyond a general discussion of key findings, and we cannot confirm with full certainty that a management plan lacks a cumulative effects provision, within or in a separate process or document, if we located none.

We believe that it is beneficial to explicitly state, at the MPA management plan level, the intent to incorporate CE into ongoing MPA management. Effective MPA management must be adaptive, whereby new information acquired through monitoring is continually used to update management approaches (e.g., Holling, 1978; Gregory et al. 2012), and understanding the full extent of human pressure on an MPA is a critical prerequisite for a well-designed and fully functioning adaptive management cycle. Understanding cumulative effects, therefore, is critical for interpreting the ecological outcomes of protection, evaluating MPA management effectiveness, and using MPAs as reference areas for assessing the effects of global and regional-scale pressures in marine ecosystems that are not well managed, not yet managed, or cannot be managed, such as climate change or diffuse pollution (Dunham et al. 2020). When the intent to identify and assess changes in existing human activities and proposed new activities and their cumulative effects on conservation objectives is not explicitly stated in MPA management plans, MPA managers might not incorporate the indicators necessary to assess such effects into monitoring plans and/or fail to reflect them in management targets and thresholds (Bryce and Hunter, 2024). This failure compromises MPA performance assessments and misses out on the opportunity to evaluate MPAs as mitigation and adaptation tools.

### Cumulative effects in action

**Fig. 4:**
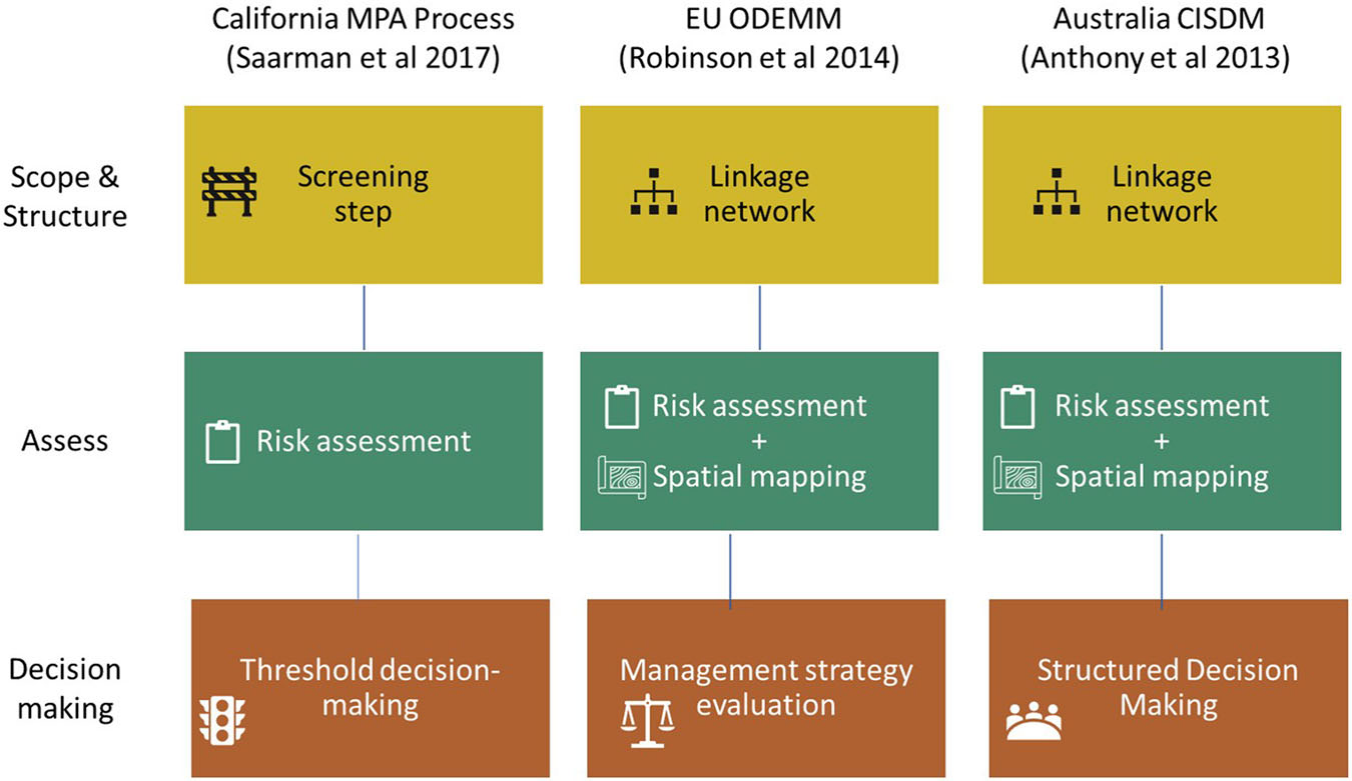
Structure of three cumulative effects management projects that include all three steps of the process, Scope and structure, Assessment and Decision-making

Our review of the primary and grey literature demonstrates a growing knowledge base to support cumulative effects management in MPAs. At present, however, only a handful of existing applications in MPA contexts include all three components of the CE framework: scope and structure, assessment and decision-making (Anthony et al. 2013; Furlan et al. 2020; Robinson et al. 2013; Saarman et al. 2017). Cumulative effects questions are embedded in complex social-ecological systems, making multiple, methodologically diverse assessments a necessity (Willsteed et al. 2018). In order to carefully consider cumulative effects in MPAs, practitioners can build tailored processes based on resources required and timeframe for decision-making in their specific circumstances. We identified methods from several studies which have the potential to form components of such processes. However, not all pieces can be combined interchangeably. Here we highlight three applications encompassing the full process, from scope to assessment and decision-making, so that MPA practitioners can visualize the possibilities (Fig. 4).

In the California MPAs, scoping for what will be included in the assessment happens in the first step in the framework, determining whether or not the proposed project, including all scientific activities, is appropriate to consider permitting within an MPA (Saarman et al. 2017). The assessment step is a quantitative ecological decision-making framework to estimate potential cumulative impacts of scientific research activities proposed for MPAs to guide managers with informed permitting decisions. The decision component consists of an impact threshold comparison to determine if the estimated cumulative impacts of all projects exceed policy-based acceptable impact thresholds for species, assemblages, or habitats.

ODEMM, an ecological risk assessment approach from the European Commission, consists of two components: a linkage network with sector-pressure-ecosystem component linkage chains (Robinson and Culhane, 2020), followed by a pressure assessment where each individual linkage chain is scored on five attributes: spatial overlap, frequency of occurrence, degree of impact, resilience and persistence (Borgwardt et al. 2019; Robinson et al. 2013). Scoring is consensus-based and completed by a combination of expert opinion and literature review. The results allow a relative ranking of the sectors, pressures, or ecological components in the assessment using summary statistics such as the average or sum of scores (Robinson et al. 2014; Robinson et al. 2013). The decision-making Integrated Management Strategy Evaluation (iMSE) examines how linkage chains (based on Driver-Pressure-State combinations) contribute to the risk of not fulfilling policy objectives, with a reduction in ecological risk associated with a management action reflecting its effectiveness (Robinson et al. 2014).

In Australia, the Great Barrier Reef World Heritage site is managed using a comprehensive framework, the ‘Cumulative Impacts Supporting environmental Decisions Model’ (CISDM) model, that combines qualitative models to identify Driver-Activity-Pressure-Impacts linkages (Anthony et al. 2013). Qualitative models are developed in workshops with scientific experts and then transformed to probabilistic ecosystem models using Bayesian networks (BN). Cumulative effects are assessed by analyzing these models together with zones of influence (ZOI) to calculate estimated risk. This information is used with two structured decision-making phases to examine potential impacts of development proposals (Anthony et al. 2013). In an adaptation of the CISDM, qualitative models are replaced with an assessment that combines risk and spatially-explicit methods, the Spatial Cumulative Impacts Risk Analysis (SCIRA) model, a dynamic spatial predictive model of cumulative impacts (Uthicke et al. 2016). SCIRA uses dynamic environmental layers as input variables and bathymetry, land, and habitat masks as base layers designed to be incorporated into the CISDM model (Anthony et al. 2013). The differences between the risks associated with different scenarios allows managers to directly compare alternative management options.

## Embedding Cumulative Effects in MPA Management

**Fig. 5:**
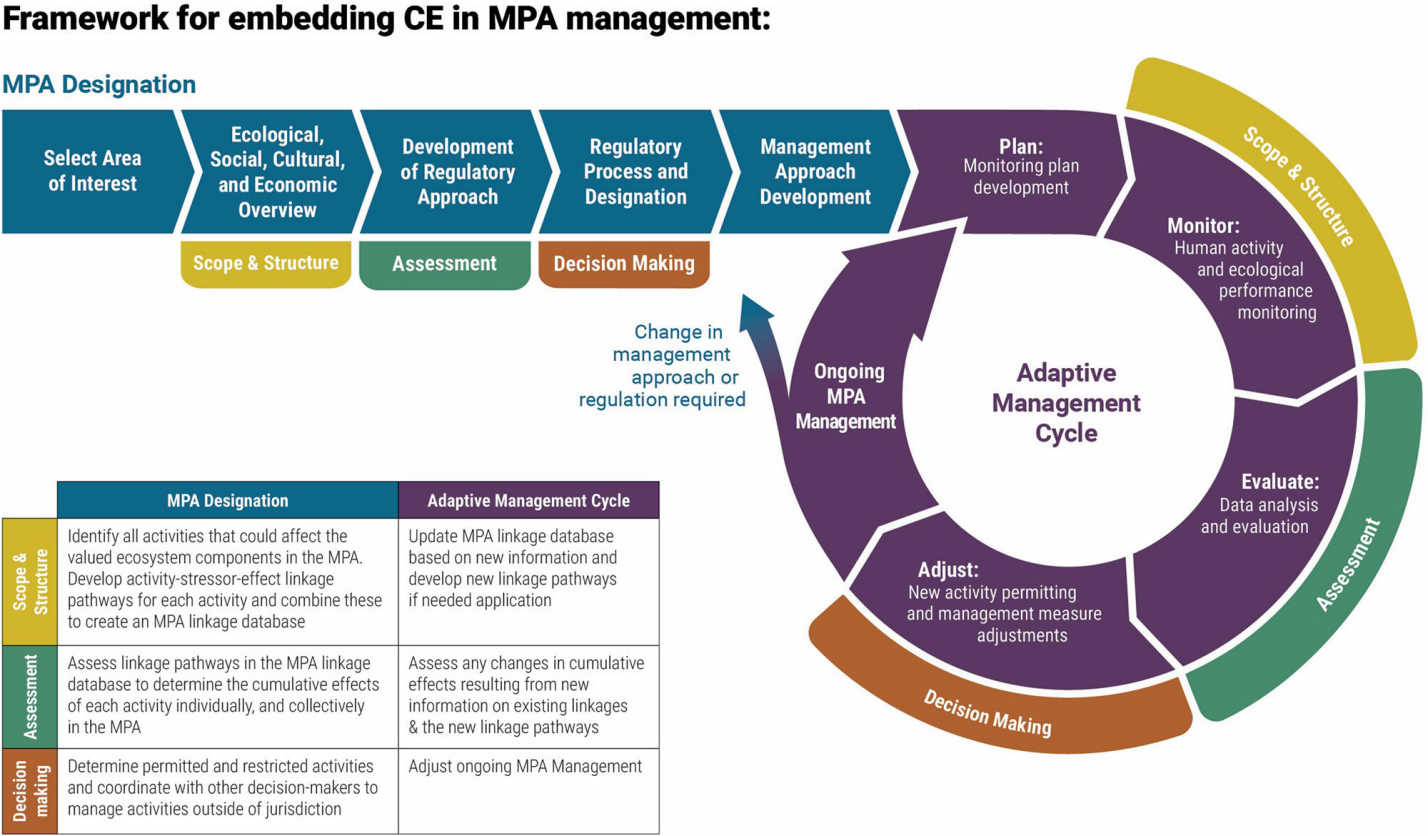
Cumulative effects steps, Scope & Structure (yellow), Assessment (green), and Decision Making (orange), embedded into the MPA designation process and the ongoing adaptive management cycle. The generalized approach for the designation and adaptive management cycle was adapted from Canada’s approach to MPA creation (DFO, 1999)

**Fig. 6:**
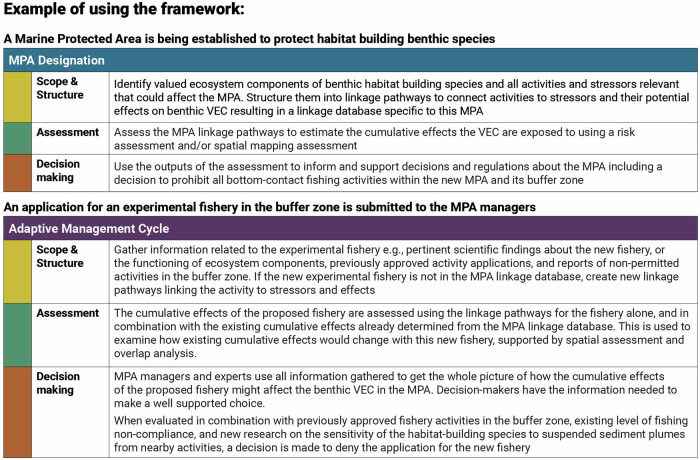
Using the CE framework: A hypothetical example MPA protecting benthic biogenic habitat and associated biodiversity and how the steps are applied in practice in the MPA designation and adaptive management cycle. Cumulative effects steps are color-coded, Scope & Structure (yellow), Assessment (green), and Decision Making (orange), as in Fig. 5. VEC refers to Valued Ecosystem Component.

Recognition that MPA conservation objectives are influenced by multiple stressors that evolve and interact with one another in both space and time— the CE mindset—is crucial for meaningful, fit-for-purpose MPA designation and management (Sinclair et al. 2017; Willsteed et al. 2018). To meet MPA management challenges in the face of increasing anthropogenic stressors, the steps of the cumulative effects framework need to be applied, ideally, first in the designation process and then again during the ongoing management cycle, as depicted in Fig. 5. The CE steps align well with phases of a general MPA designation and management cycle (based on Canadian MPA management, DFO, 1999). *Scope and structure* can occur in the overview phase, identifying the area’s focal components and the activities and stressors affecting the area. *Assessment* can occur during the development of the regulatory approach, with a risk assessment or spatial analysis of the stressors to identify those stressors or areas with relatively high risk for potential inclusion in regulation and monitoring. The results of the assessment then help guide the identification of regulatory processes and policy instruments required. *Decision Making* occurs in the regulatory process phase where the legislation is applied and the area formally designated. The CE steps should be applied again during adaptive management, or for the first time for MPAs that were designated prior to consideration of cumulative effects. In the adaptive management cycle, *Scope and structure* occurs during ongoing monitoring of components as well as activities and stressors. *Assessment* can occur during the evaluation phase, and *Decision Making* during the adjustment phase (Fig. 5). In order to further illustrate how the CE steps can be embedded, we offer a hypothetical example of an MPA established to protect benthic habitat built by filter feeding species (Fig. 6).

Despite the broad acknowledgement of their importance, the consideration of cumulative effects is seldom implemented in management applications (Hollarsmith et al. 2022; Willsteed et al. 2023) and we found little evidence of their practical inclusion in MPA projects. The global database of MPA management plans showed increasing inclusion of CE language over time, however a practical application of all steps of the cumulative effects framework remains the exception. Our synthesis of the growing primary and grey literature offers a tool for practitioners to overcome a major limitation: to identify, gather and synthesize relevant cumulative effects knowledge in order to choose appropriate methods and the approaches to apply them in MPA management.

Given the complexity of the issue and diversity of methods and studies available, the disparity between the legal requirements to consider cumulative effects and actual implementation in our review of the literature and MPA management plans, was not unexpected. The framework for embedding CE into MPA management is one tool that MPA managers can use as a blueprint to bridge this gap, providing an overall structure to include cumulative effects in MPA management. The level of detail and depth required is dependent upon the properties of the protected ecosystem, MPA characteristics, available resources, and other jurisdiction- and location-specific factors. We still have a lot to learn about the real-world implementation of these methods, and this knowledge will continue to grow with the continued application and documentation of case studies globally.

MPA designation can be a lengthy process, while permitting and impact assessment often have short decision timeframes. As such, the development and use of evergreen records like an MPA linkage database and assessments that only need to be updated with new information makes good use of resources long term (Hannah et al. 2020). Common resources, like an open access linkage database, built using a standard lexicon, would require dedicated funding for long term maintenance and support, likely by governmental organisations, and would benefit from the inclusion of diverse types of evidence from sources including Indigenous knowledge, government, industry, and academia (Murray et al. 2020; Cannon et al. 2024).

Adopting a CE mindset, leveraging available methods, and further developing customized CE scoping, assessment, and decision-making methods for each MPA are paramount. Our framework illustrates how to embed CE practices firmly within an MPA management cycle, with examples from a Canadian perspective, in both the designation phase and adaptive management cycle, and supports comprehensive scoping, meaningful assessments, and clear and transparent decision-making in MPAs globally.

## Supplementary information

Supplementary material

## Data Availability

No datasets were generated or analysed during the current study.
